# Global research on endocrine disruptors as emerging hazards for human health and the environment

**DOI:** 10.3389/fendo.2025.1561711

**Published:** 2025-06-26

**Authors:** Doris Klingelhöfer, Markus Braun, Janis Dröge, Dörthe Brüggmann, David A. Groneberg

**Affiliations:** Institute of Occupational, Social and Environmental Medicine, Goethe University Frankfurt, Frankfurt, Germany

**Keywords:** bisphenol A, endocrine diseases, neurological diseases, pharmaceuticals, estrogen, thyroid hormones

## Abstract

Endocrine disruptors (EDs) contaminate nearly every ecosystem and are significantly associated with different neurological and neurodevelopmental disorders. To date, there is no comprehensive literature on global publication efforts. Since there are many unknown substances, modes of action, and risks of EDs, it is necessary to provide detailed insight into global publication patterns from temporal, regional, and socioeconomic perspectives. Hence, this review article provides background information for all stakeholders, from scientists to clinicians and policymakers. A disproportionate increase in research activity was observed, mainly from the USA and China, with a strong north-south divide. Multi-disciplinarity is characteristic, with a trend toward an ecological focus. Low- and middle-income economies are underrepresented in research on EDs. Therefore, global research needs to be refocused and expanded to more global approaches that take inspiration from the few successful collaborations with their synergistic effects.

## Highlights

- A disproportionate increase in research activity is shown.
- Global research is multidisciplinary and increasingly tending towards ecological foci.
- There is a strong north-south divide in publication performance at the global level.
- The dominant research countries are the USA and China.
- Regulations must be established, and the risks must be made public.

## Introduction

In just a few decades, the scientific issue of endocrine disorders has undergone a remarkable transformation from an entirely obscure topic to a common concern. There is a close interaction between the brain and the endocrine system. The brain is a major target for hormones such as steroids, thyroid hormones, or growth hormones. Therefore, the influence of *endocrine-disrupting chemicals* (EDCs) significantly impacts brain function and behavior by neuronal remodeling or neuroplasticity (1).

EDCs have been found in every ecosystem tested, even in the most remote areas of the world (2). EDCs are naturally occurring substances or synthetically produced chemicals and are now considered emerging contaminants and a global public health challenge. As an umbrella term for EDCs, *endocrine disruptors* (EDs) also include physical agents such as artificial light (3) or radiation (4) that affect the endocrine system and impair human and animal health in various ways. The WHO (*World Health Organization*) further defines EDs as substances that “lead to endocrine disruption in an intact organism, its progeny, or (sub)populations” (5, 6). Many products have been identified as containing EDs, including pesticides, cosmetics, household cleaners, plastic containers, fabric, upholstery, electronics, and medical equipment (7). EDs also include industrial or agricultural substances, drugs, e.g., heavy metals, dioxins, PCBs, pesticides, phthalates, Bisphenol A (BPA), Lithium as an antipsychotic, or anticancer drugs such as Sunitinib (6, 8–10). They contaminate the environment through the flow of air or water and can be found even in remote places such as the Arctic (11). The environmental compartments most affected are surface waters, as EDs are mainly introduced through erosion, wastewater, precipitation, or leaching (5). Human exposure occurs through diet, inhalation, or skin contact due to environmental contamination through water, soil, or air (12, 13). Exposure of the fetus occurs via the placenta (14) or through breastfeeding (15). EDs alter the function of the endocrine system (7, 16), e.g., by interfering with the synthesis, secretion, and transport of hormones or by mimicking the action of hormones (17, 18). EDs have also been shown to disrupt thyroid function. Estrogens and thyroid hormones play a central role in brain development. They regulate metabolism on a cellular and organismic level (19). Also, the maternal brain is influenced by EDs, which can lead to maladaptive maternal behavior (20). In addition to the effects on brain development, EDs can also have negative effects on fertility, pregnancy, and sperm quality, as well as on other organ functions, such as the urogenital tract (21).

Although many adverse effects on humans and the environment have been demonstrated, the difficulty in quantifying exposure and the influence of confounding factors make it difficult to establish a causal relationship between specific EDs and disease. Most studies on neuroendocrine disorders focus mainly on the effects of estrogens, androgens, and thyroid hormones. The disruption of other endocrine signaling pathways by environmental chemicals is still poorly understood (1). In addition, toxicity studies focus primarily on acute exposure, although EDs are constantly present and thus cause chronic exposure (22). In addition, the chemicals are usually analyzed individually, although in nature they occur in many combinations (23).

Despite the growing interest in the mechanisms and effects of EDs (12), there is little knowledge about the function and the associated species-specific differences in sensitivity and the resulting lack of internationally accepted and valid test methods (24).

For future cross-regional and targeted approaches, it is essential to know the incentives and barriers of past research. Therefore, this study aims to provide background information on the chronological and geographic characteristics of ED research output related to human health and environmental risks. To achieve this, a thorough analysis of publications on ED was conducted, assessing the main actors of research and their networking in combination with socio-economic and pollution parameters, so that the drivers and barriers to research engagement could be uncovered.

## Methods

### Methodological platform

The methodology used for this study is based on the *New Quality and Quantity Indices in Science* (NewQIS) bibliometric platform (25, 26), which was developed to provide global publication patterns of research fields, taking into account chronological and geographical aspects. The default data source is the online database Core Collection from *Web of Science* (WoS).

### Creation of the database

The thorough elaboration of an adequate search string in WoS is essential for the creation of a valid and representative database for the evaluation of research on EDs. Therefore, the search string combines synonyms and compound terms that relate precisely to the topic and allows searching via the TOPIC search tool of WoS, in addition to the title, abstract, and keywords.

After retrieving the entries found in the described way, they were filtered by the document type “article” to include only original research publications in the analysis. The metadata were stored in an MS Access database and sorted according to the different analysis parameters. Additionally, several parameters had to be corrected and standardized manually. This was the case regarding the authors’ institutions due to varying denominations. Also, the countries of origin had to be updated by comparing them with a current list of countries.

### Analysis parameters

To assess temporal influences and patterns on ED research, the annual number of articles, citations, average citation rate (number of citations/number of articles), and number of collaborative articles per year were analyzed. In addition, the most frequently cited articles were identified and assigned to their year of publication to assess their influence on global research activity. The purpose of determining national publication performance was to identify key players and to calculate the relative share of the most published countries at 4-year intervals from 1998 to 2022. In addition to article counts, citation counts, and citation rates were also determined for each country. Socioeconomic and scientific infrastructure indices of countries with at least 30 articles on ED (analysis threshold) were included as ratios of publication numbers to provide a more accurate picture of the international research landscape. For this purpose, the number of articles of the countries was set in relation to the population size, the *Gross Domestic Product* (GDP), the number of researchers, and the *Gross Expenditure on Research and Development* (GERD). In addition, a proxy for the generation of EDs per country was determined. The number of items in the countries was set in relation to the plastic waste generation per country (R_PW_ = number of items/plastic waste in 1000 t/day). The data used are from 2010 (27, 28). Plastic waste was used as a key indicator for the industry with the most ED emissions (especially BPA), namely the packaging industry.

International partnerships and collaborations of institutions were identified, and networks were mapped. Some geographic results were presented using *Density Equalizing Map Projections* (DEMPs) (29). These distort the country sizes according to the size of an assessment parameter based on an equal parameter density of all countries. An exception is Antarctica and the oceans, which are given an average value of the analysis parameter to obtain the gross structure of the world map.

## Results

The created database contains 19,099 articles (n) related to ED.

### Chronological development

**Figure 1:**
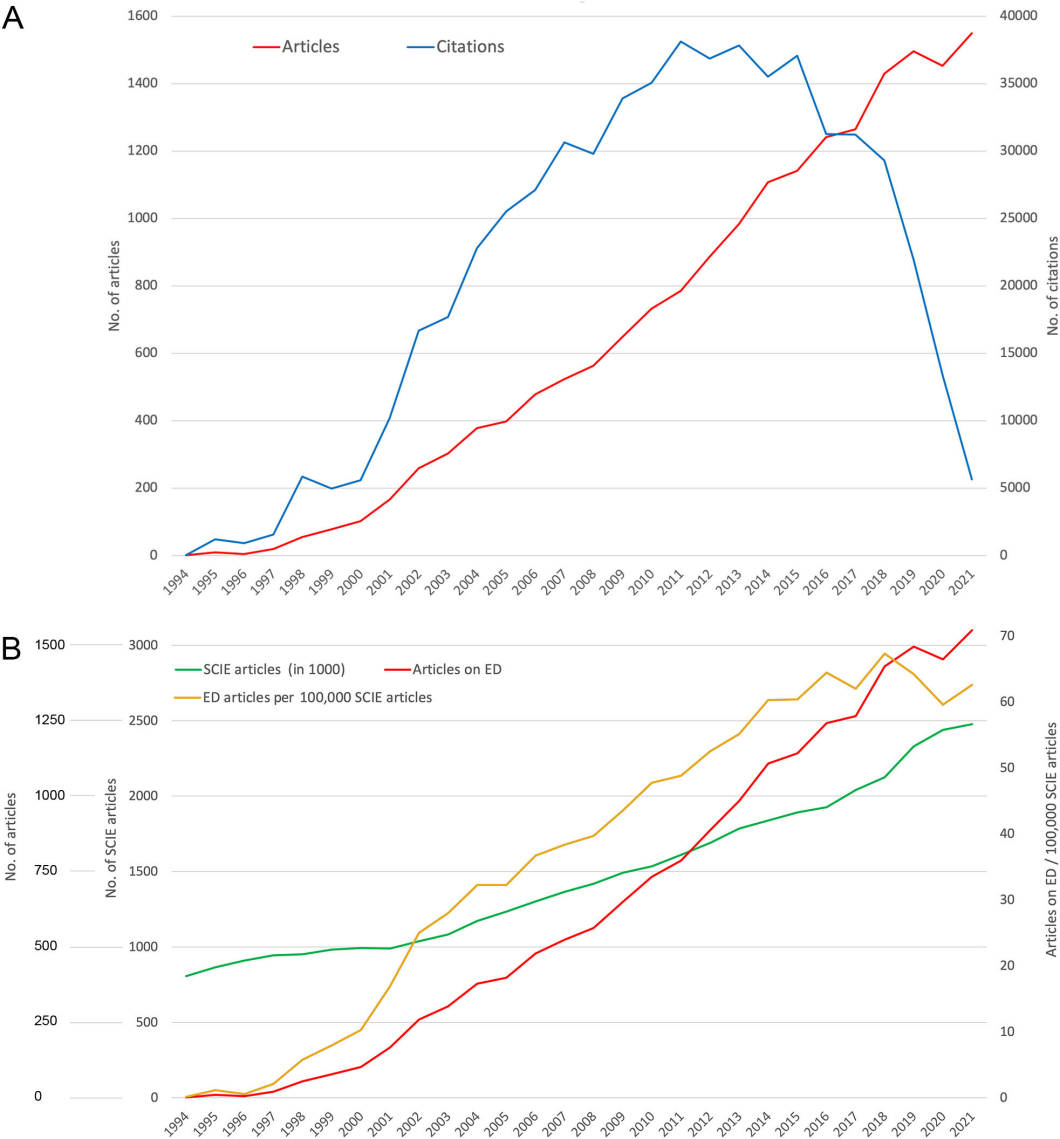
Development of publication values on endocrine disruptors (EDs) over time. **(A)** Number of articles and number of citations. **(B)** Number of articles, number of all articles listed in the Science Citation Index Expanded (SCIE articles in 1000) of Web of Science (WoS), number of ED articles per 100,000 SCIE articles.

The articles in the database range from 1994 to 2022. The initially low number of articles increased slightly after 1997 and significantly from 2000 onward (**Figure 1A**), as evidenced by relatively greater interest in ED research compared with the total number of articles listed in the *Science Citation Index Expanded* (SCIE) of WoS (**Figure 1B**).

This ratio decreased in 2018, when the number of ED articles per SCIE articles fell.

The development of the articles shows a strong upward trend, which, except for the last few years, even exceeds the development of all SCIE articles. Likewise, the number of citations increases above average until the maximum value is reached in 2011 (c = 38,131). Until the average citation half-life (about 8 years for biomedical articles) in 2015, the curve of average citation numbers decreased only slightly and then rapidly until today. Before this, two small interim citation peaks can be identified: 1998 (c = 5864) and 2007 (c = 30,650).

The most-cited articles on ED with the publication years are listed in **Table 1**.

**Table 1:** Most-cited articles on endocrine disruptors (EDs).

Author (Countries of origin)	Year	Citations	Title	Journal
Anway, MD et al. (USA) (47)	2005	1750	Epigenetic transgenerational actions of endocrine disruptors and mate fertility	Science
Jobling et al. (UK) (48)	1998	1516	Widespread sexual disruption in wild fish	Environmental Science & Technology
Benotti et al. (USA) (49)	2009	1180	Pharmaceuticals and Endocrine Disrupting Compounds in US Drinking Water	Environmental Science & Technology
Thomas et al. (USA) (50)	2005	1074	Identity of an estrogen membrane receptor coupled to a G protein in human breast cancer cells	Endocrinology
Westerhoff et al. (USA) (51)	2005	1061	Fate of endocrine-disruptor, pharmaceutical, and personal care product chemicals during simulated drinking water treatment processes	Environmental Science & Technology
Kasprzyk-Hordern et al. (UK) (52)	2009	1033	The removal of pharmaceuticals, personal care products, endocrine disruptors and illicit drugs during wastewater treatment and its impact on the quality of receiving waters	Water Research
Heudorf et al. (Germany) (53)	2007	977	Phthalates: Toxicology and exposure	International Journal of Hygiene & Environmental Health
Kim et al. (USA, South Korea) (54)	2007	964	Occurrence and removal of pharmaceuticals and endocrine disruptors in South Korean surface, drinking, and waste waters	Water Research
Lang et al. (UK, USA) (55)	2008	945	Association of urinary bisphenol A concentration with medical disorders and laboratory abnormalities in adults	JAMA
Dolinoy et al. (USA) (56)	2007	904	Maternal nutrient supplementation counteracts bisphenol A-induced DNA hypomethylation in early development	PNAS

### Countries’ publication output

Of the entire database (n = 19,099), almost all articles (n = 19,044, 99.71%) could be assigned to a country of origin. These articles were included in the geographic analyses.

**Figure 2:**
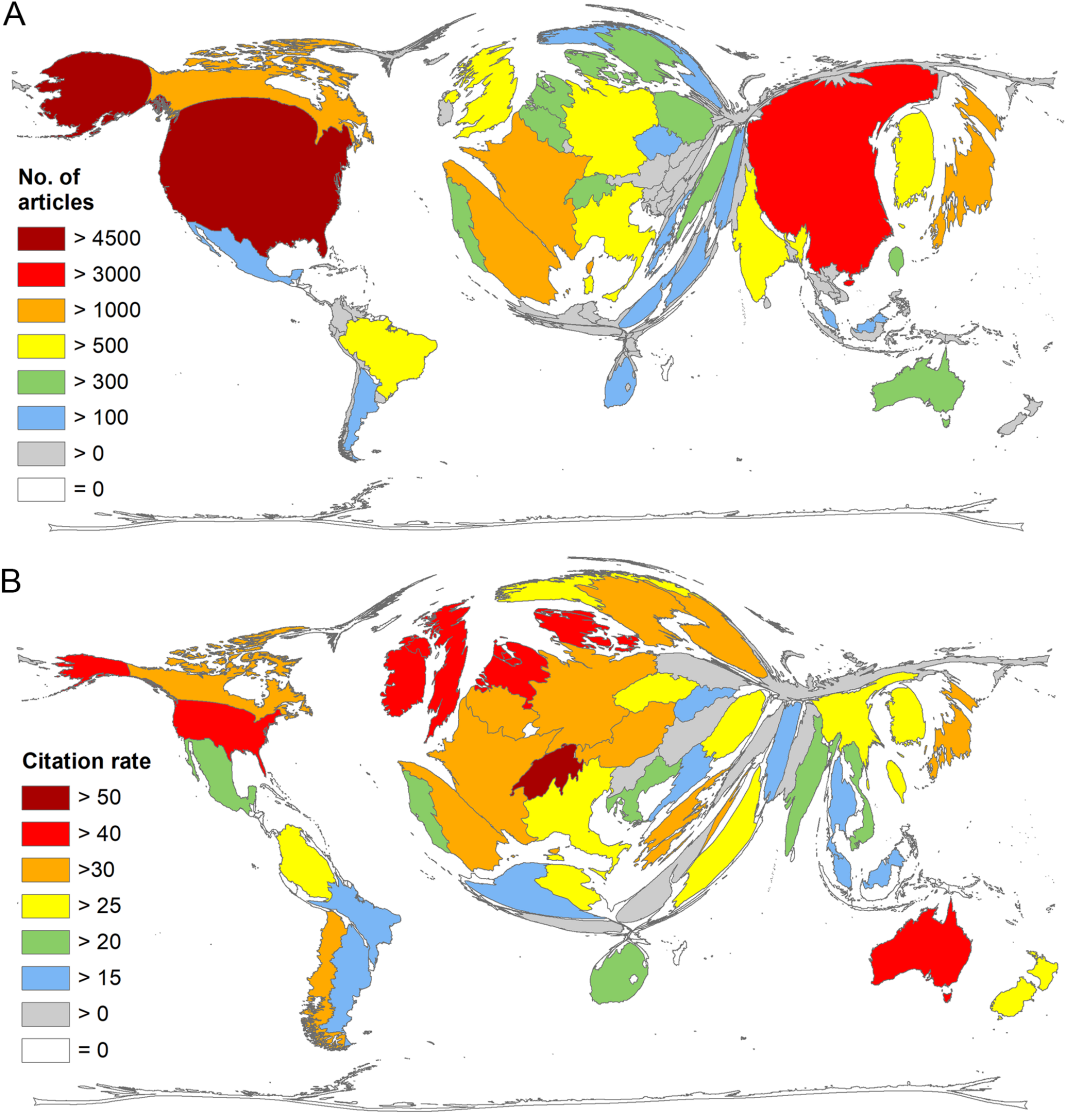
National publication output on endocrine disruptors (ED). **(A)** Number of articles per country. **(B)** Average citation rate (number of citations/number of articles) per country, analysis threshold: 30 articles on ED.

The major contributors to ED research were the USA (n = 4717) and China (n = 3634). More than 1000 articles were contributed by Japan (n = 1326), Spain (n = 1164), France (n = 1049), and Canada (n = 1002) (**Figure 2A**). The ranking for the number of citations is similar to that for the number of articles, except the UK, which moved up to 5th place. In terms of average citation rate (cr), Switzerland leads the ranking (cr = 50.28), followed by Denmark (cr = 47.71), Ireland (cr = 46.23), the UK (cr = 42.53), and the Netherlands (cr = 42.11) (**Figure 2B**).

**Figure 3:**
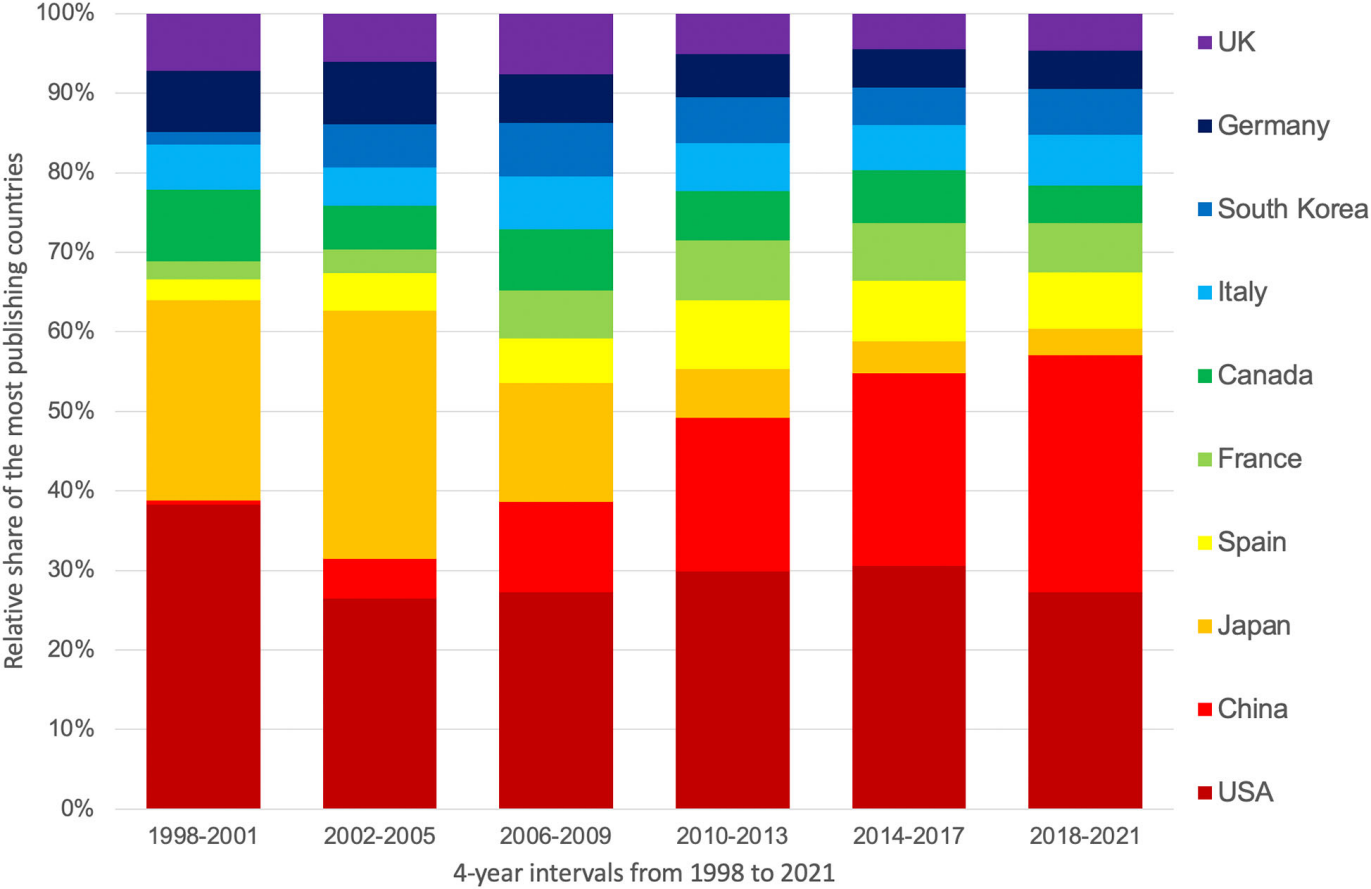
Change in the relative share of the most publishing countries at 4-year intervals from 1998 to 2021.

To assess trends in national involvement in ED research, 4-year periods from 1998 to 2021 (**Figure 3**) and the year 2022 were analyzed. While China’s contribution increased significantly over time (0.51%-29.83%), the share of US (38.30%-27.25%) and Japanese articles decreased. In the case of Japan, this decline was significant, falling from 25.19% to 3.27%. In 2022, the top 5 ranking by the time of the evaluation is China (n = 305), followed by the USA (n = 167), India (n = 60), Spain (n = 58), and Brazil (n = 53).

### Publishing institutions

The scientific institutions publishing most frequently on ED are listed in **Table 2**.

**Table 2:** The institutions that publish most frequently on endocrine disruptors (EDs).

Institution	Country	Articles	Citations	Citation rate
Chinese Academy of Science (CAS) - Government	China	507	17077	33.68
National Institutes of Health (NIH) - Government	USA	313	12201	38.98
US Environmental Protection Agency (EPA) - Government	USA	304	14740	48.49
Spanish National Research Council (CSIC) - Government	Spain	302	13833	45.80
Harvard University	USA	247	9959	40.32
US Center for Disease Control & Prevention (CDC) - Government	USA	177	10313	58.27
University Copenhagen	Denmark	172	7832	45.53
Nanjing University	China	157	3655	23.28
University Granada	Spain	155	5300	34.19
University Florida	USA	137	5067	36.99

### International collaboration

**Figure 4:**
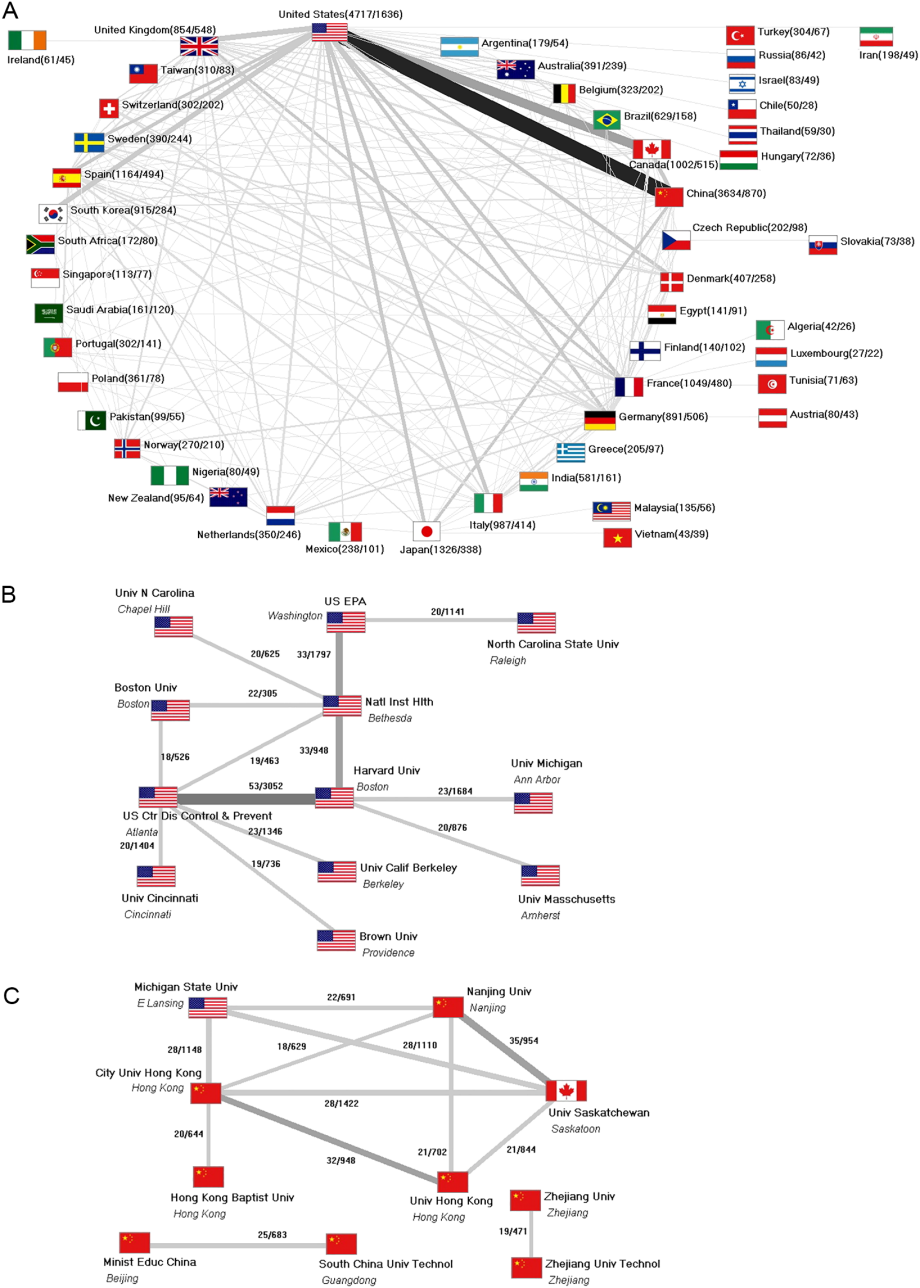
Scientific collaborations. **(A)** Countries (display threshold: 10 collaboration articles). **(B)** US-American institutions (display threshold: 18 collaboration articles), numbers in brackets (number of articles/number of collaboration articles), number on connecting lines (number of collaborations/number of citations). **(C)** Chinese institutions (display threshold: 18 collaboration articles), numbers in brackets (number of articles/number of collaboration articles), number on connecting lines (number of collaborations/number of citations). Univ, university; US EPA, U.S. Environmental Protection Agency; Natl Inst Hlth, National Institutes of Health; US Ctr Dis Control & Prevent, U.S. Center for Disease Control & Prevention. **(C)** Chinese institutions. Technol, Technology; Minist Educ China, Ministry of Education China.

A total of 4370 articles were published as international collaborations (22.88%). Most of these were bilateral partnerships (n = 3386, 77.48%). A broad international network has been established, with the USA and China cooperating the most (**Figure 4A**). At the institutional level, two networks can be identified that have formed between US institutions on the one hand (**Figure 4B**) and Chinese institutions, some in collaboration with an institute from the USA or Canada on the other (**Figure 4C**).

### Influence of socio-economic characteristics and scientific infrastructure

Including the socioeconomic characteristics of the countries and the scientific infrastructure as quotients between the number of articles on ED and the respective parameters leads to different rankings (**Table 3**).

**Table 3:** Top 10 countries of the socio-economic and scientific infrastructural analyses (threshold: 30 articles on endocrine disruptors).

Socio-economic indices							
Country	Articles	Population (mill.)	R_POP_	Country	Articles	GDP (1000 bn US-$)	R_GDP_
Denmark	407	5.75	70.76	Tunisia	71	0.04	1667.83
Norway	270	5.34	50.58	Slovenia	79	0.05	1459.26
Sweden	390	9.97	39.11	Portugal	302	0.24	1246.93
Slovenia	79	2.08	38.02	Denmark	407	0.36	1140.56
Switzerland	302	8.53	35.42	Serbia	50	0.05	987.35
Portugal	302	10.26	29.45	Greece	205	0.21	967.23
Belgium	323	11.48	28.13	Spain	1164	1.42	819.55
Canada	1002	37.08	27.03	Czech Rep.	202	0.25	811.41
Finland	140	5.52	25.35	Sweden	390	0.56	702.13
Spain	1164	46.69	24.93	Slovakia	73	0.11	691.54

Indices of scientific infrastructure							
Country	Articles	Researcher (1000)	R_RES_	Country	Articles	GERD (bn US-$)	R_GERD_
Denmark	407	43.92	9.27	Tunisia	71	0.31	232.16
Spain	1164	140.12	8.31	Serbia	50	0.47	107.45
Norway	270	34.34	7.86	Portugal	302	3.27	92.40
Slovenia	79	10.07	7.85	Slovakia	73	0.89	82.36
Sweden	390	56.80	6.87	Greece	205	2.57	79.69
Italy	987	152.31	6.48	Egypt	141	1.81	78.00
Portugal	302	47.65	6.34	Slovenia	79	1.05	74.97
Mexico	238	39.19	6.07	Argentina	179	2.59	68.99
Canada	1002	167.44	5.98	Spain	1164	17.64	65.98
So. Africa	172	29.11	5.91	Colombia	68	1.04	65.15

GDP, Gross Domestic Product; GERD, Gross Expenditures in Research & Development. FTE, Full-Time Equivalents; R_GDP_, number of articles/Gross Domestic Product in 1000 billion US-$; R_POP_, number of articles/population size in million inhabitants; R_GERD_, number of articles/Gross Expenditures for Research & Development in billion US-$; R_RES_, number of articles/number of researchers in 1000 full-time equivalents.

**Figure 5:**
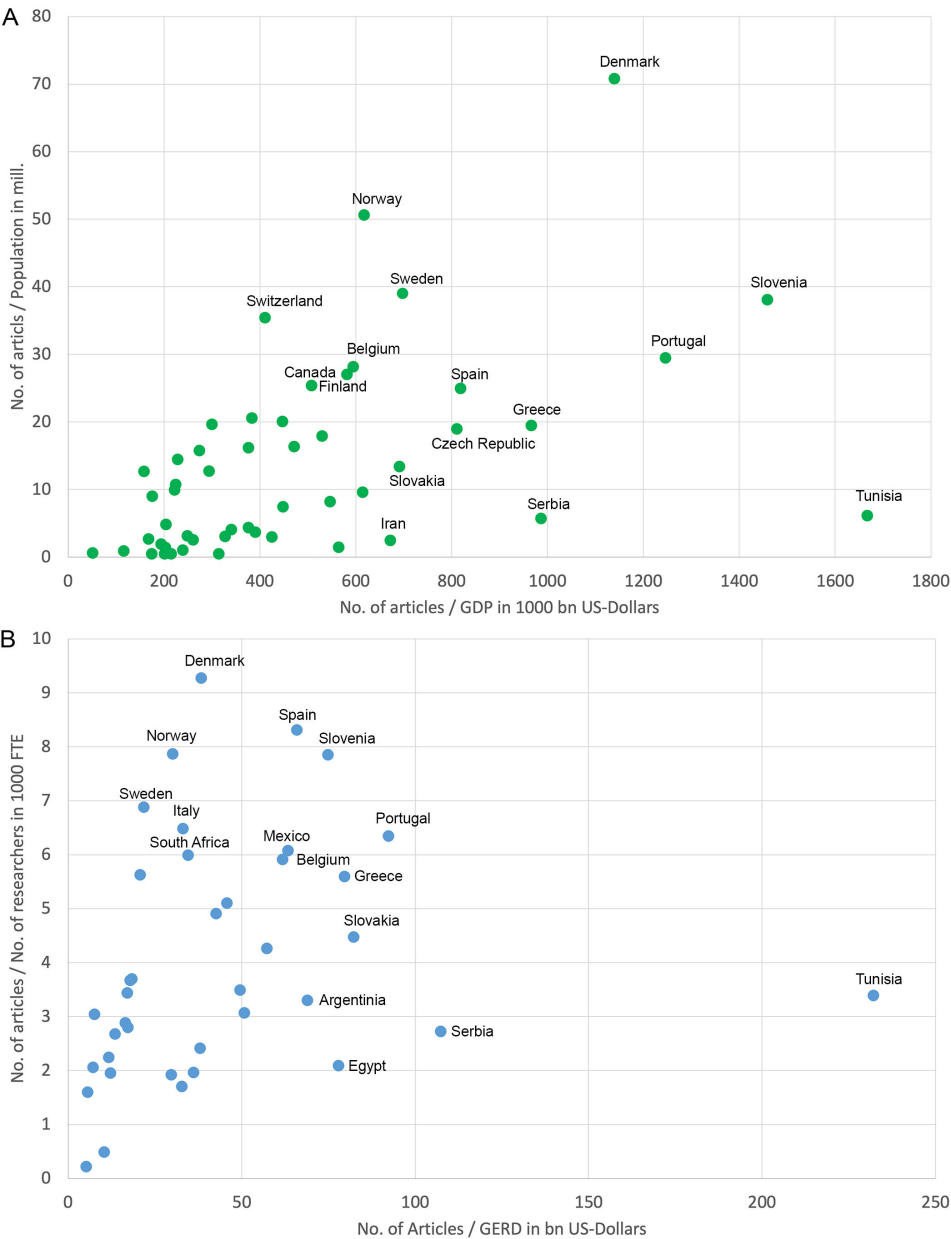
Socioeconomic and demographic ratios (threshold: 30 items). The 10 best-performing countries for each parameter are indicated with country names. **(A)** Ratio between the number of articles and population in millions of inhabitants and the ratio between the number of articles and gross domestic product (GDP) in US$1000 billion. **(B)** Ratio between the number of articles and the number of researchers in 1000 full-time equivalents (FTE) and the ratio between the number of articles and gross expenditure on research and development (GERD) in billions of US dollars. Colombia (ranked 10th in R_GERD_) could not be displayed due to the lack of researcher numbers.

**Figure 5A** shows the countries' positioning in terms of the ratios between the number of articles and the population size (y-axis) and between the number of articles and GDP (x-axis). **Figure 5B** shows the countries' ratios between the number of articles and the number of researchers (y-axis) and between the number of articles and the GERD (x-axis).

### Plastic waste generation per country

**Figure 6:**
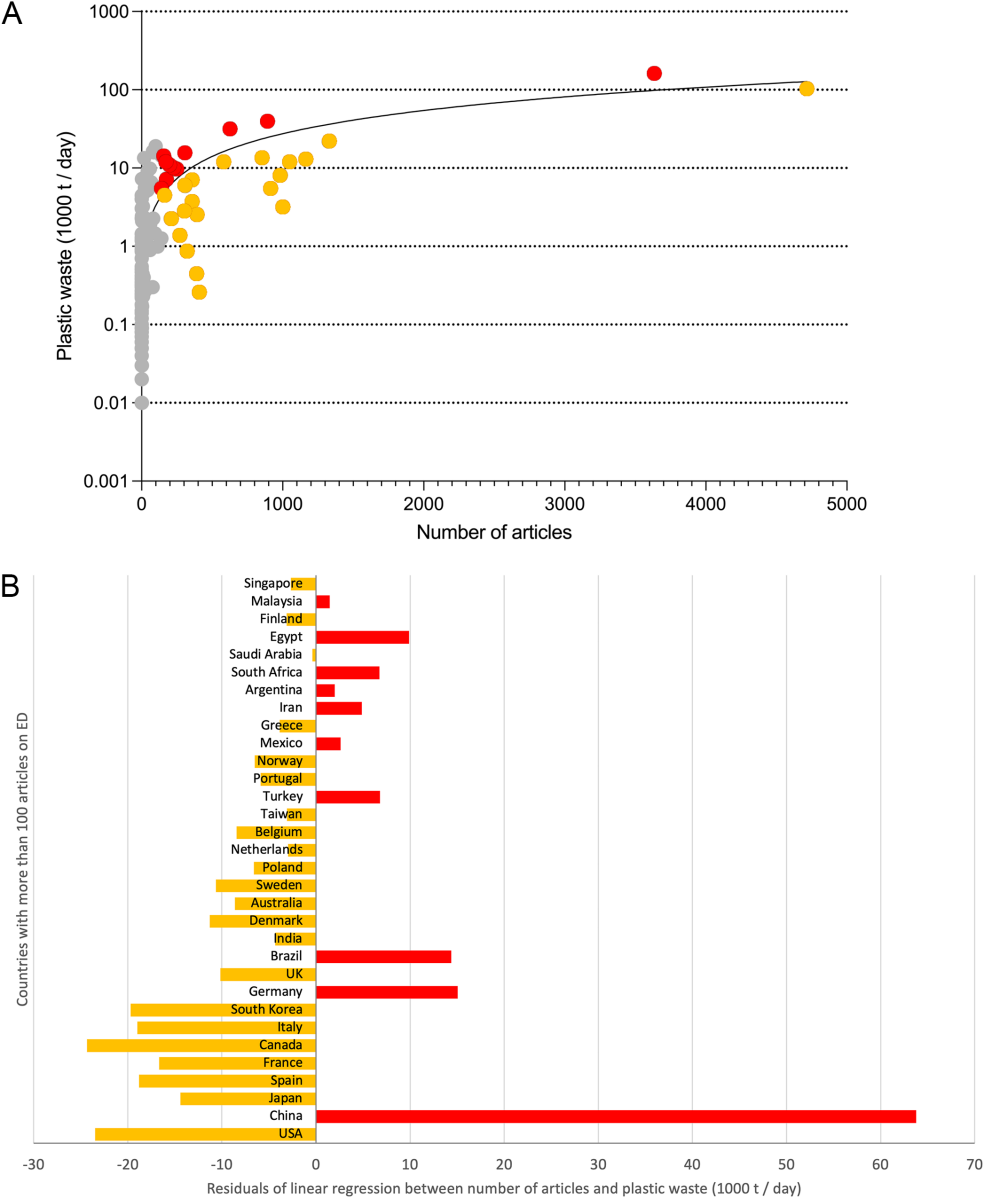
Linear regression between number of articles and plastic waste generation in 1000 t/day, red dots: Deviation from the regression line to the disfavor of the article numbers, yellow dots: deviation from the regression line in favor of the article numbers, gray dots: countries with less than 100 articles on ED. **(A)** Linear regression (Spearman), y-axis: logarithmic. **(B)** Residuals for countries with more than 100 endocrine disruptor (ED) articles, sorted by ascending number of articles, (presentation threshold: 100 articles on ED per country.

In the absence of data on ED generation per country, a proxy was used for the analysis. *Plastic waste generation* (PWG) is appropriate for this purpose, as the plasticizer BPA is one of the most treated EDCs in our research and represents plastic packaging as one of the most emitting industries of EDCs (30). Linear regression analysis shows a significant correlation between PWG and the number of articles per country (p<0.0001, Spearman) (**Figure 6A**), but no correlation between PWG per capita and publication output.

The plot of the residuals of the linear regression shows the deviations in favor (yellow) and disfavor (red) of the publication output of the countries to EDs (**Figure 6B**). Relatively lower contributions were made by China, Germany, and Brazil, which are at the top of the publishing countries. The USA, Japan, and the other European countries that publish a lot on EDs showed a better ratio between articles and plastic waste.

The corresponding ratios for the countries with at least 30 articles on ED (threshold) (R_PWG_ = number of articles on ED/Plastic waste generation in 1000 t/day) are led by Denmark (R_PWG_ = 1574.47), followed by Sweden (R_PWG_ = 864.36), Belgium (R_PWG_ = 373.84), Canada (R_PWG_ = 315.84), and Slovenia (R_PWG_ = 259.44) (**Table 4**).

**Table 4:** Countries with the highest R_PWG_ = ratio of number of articles to plastic waste generation (1000 t/day), data from 2010 (analysis threshold: 30 articles on ED).

Country	Articles	Plastic waste (1000 t/day) in 2010	R_PW_
Denmark	407	0.26	1574.47
Sweden	390	0.45	864.36
Belgium	323	0.86	373.84
Canada	1002	3.17	315.96
Slovenia	79	0.30	259.44
Norway	270	1.37	196.79
South Korea	915	5.48	167.07
Australia	391	2.51	155.85
Italy	987	8.11	121.75
Singapore	113	0.99	114.21

### Research foci

**Figure 7:**
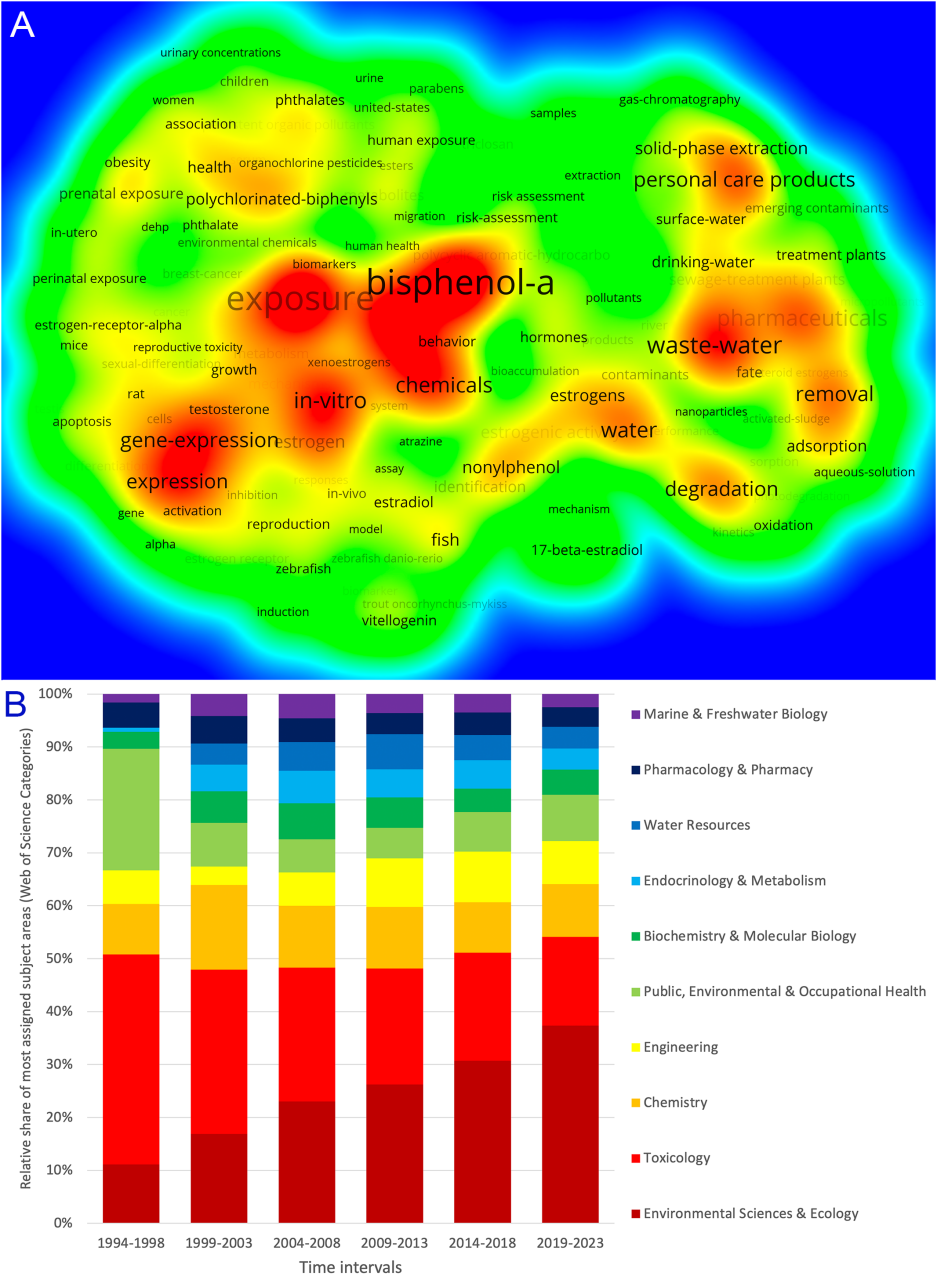
Research foci. **(A)** Density clustering of keyword (display threshold 200 occurrences). **(B)** Relative share of most assigned Web of Science categories in 5-year intervals.

Analyzing the keywords used, a density-based picture of the most focused research topics emerges (**Figure 7A**). “Bisphenol A” was the most frequently used word, with 4729 occurrences, followed by “exposure,” which occurred 2720 times. “Waste-water” was used 1580 times and formed a cluster with “personal care products” and “pharmaceuticals” as sources of EDs, and “removal” of solid and liquid phase contamination. Another topic that was frequently addressed was “gene-expression” of EDs.

The WoS categories also relate to the topics covered. The most frequently assigned category was “Environmental Science & Ecology” (n = 6579), followed by “Toxicology” (n = 4615), “Chemistry” (n = 2359), “Engineering” (n = 1841), and “Public, Environmental & Occupational Health” (n = 1636). The proportions of the categories have changed over time. For example, the category “Environmental Science & Ecology” showed a positive trend from 11.11% in the first evaluation interval (1994-1998) to 37.35% in the last interval (2019-2023), ranking first (relative percentages of the 10 most assigned WoS categories). In contrast, the relative percentage of “Toxicology” decreased from 39.68% to 16.75%. “Water resources” did not come into focus until the 2000s, with more or less equal shares of about 5% (**Figure 7B**).

## Discussion

This review article depicts the global research on all EDs that are an emerging hazard to human health, especially to neurological disorders. The first article was written by T. Colborn of the *World Wildlife Fund* (WWF) in 1994, in which he also called for greater awareness of exposure to synthetic chemicals that affect the endocrine system. He pointed out the difficulty in determining these effects, which resulted in a low scientific effort on risk assessment (31). This raised awareness, albeit only slightly, of the effects of EDs as a research topic, which began in 1995 with the published announcement by the United Kingdom (UK) to promote research on EDs (32).

The global key players are the USA and China. Overall, there is a clear North-South divide, which is typical for most research areas (26). Infrastructural and monetary sources favor the northern high-income economies in research and development (R&D).

US-American and European regulations have become the benchmark for other countries (33). However, screening and testing programs in the USA are limited only to estrogenic EDs, and EU regulations focus primarily on pesticide restriction.

However, the EU also calls for minimizing human exposure to EDCs in general and identifying substances of “very high concern”. According to the *International Panel on Chemical Pollution* (IPCP), commissioned by the UN, most developed countries and, to a lesser extent, emerging economies such as Brazil and China have taken measures. However, many regulations are only implicit because they do not address the causes of adverse health effects (33). India is also increasing its involvement in ED research so that by 2022, it will be among the top 5 countries publishing papers on ED. In other developing countries, there is virtually no regulatory framework. These characteristics reflect patterns of global publishing in absolute terms.

China underlines its second position through its general efforts to promote R&D and its publications (34). As a result, it ranks high in many research areas and sometimes even leads in terms of publication numbers. In addition, most of the international collaborations were between the USA and China, with the *Chinese Academy of Sciences* (CAS) and the *National Institutes of Health* (NIH) in the USA being the main contributors. At the institutional level, most collaborations took place between institutions from the same country. Of the major players, only China collaborates to some visible extent with the US and Canadian universities.

The number of national citations was registered analogously to the publication output, with the USA ahead of China by a large margin. Switzerland leads the ranking in terms of the citation rate, although it is neither one of the most cited countries nor does it contribute to the most cited publications. Two Federal Institutes of Technology were combined under the umbrella of the ETH Domain: ETH (*Swiss Federal Institute of Technology*) Zurich and EPFL (*École Polytechnique Fédérale* de Lausanne) in Lausanne, furthermore four research institutes. These mainly contribute to research on EDs in Switzerland. They established that ED emissions had already affected the environment and human health. Both institutes have been working on EDs since 1999, when the government decided to conduct a national research program, “Endocrine Disruptors: Significance to Humans, Animals, and Ecosystems”, to provide background information for policy regulations. The results unanimously underscore the importance of further research and long-term monitoring (35), leading to a higher number of publications with relatively high citation numbers in the future.

The Scandinavian countries rank first in the ratio of article number to population size (R_POP_). With their high research funding and available resources, as well as the operation of disease registries, the Scandinavian countries are often at the forefront of research comparisons (26).

Regarding economic ratios, Slovenia (GDP) and Serbia (GERD) ranked second after Tunisia.

They have been publishing papers on EDs since 2009. This can certainly be seen as a reaction to the EU regulations and as an initiator for the definition of scientific criteria for EDs (36). This has certainly also stimulated research in other EU countries. The relationship between R&D expenditure and scientific output has already been shown (26). This expenditure is largely dependent on a country’s economic strength. However, there has been a comparatively strong increase in expenditure in the regions of East, Southeast, and South Asia. From 2000 to 2017, China contributed 32% and South Korea and Japan 10% to global growth. The USA was responsible for 20%, the EU for 17%, and the other countries of the world for a total of 13% (37).

Research on endocrine disruptors has been multidisciplinary from the beginning. This had advantages due to synergy effects, but also disadvantages due to misunderstandings. The positions and approaches of toxicologists, endocrinologists, and chemists are very different, leading to complicated relationships. This also made it immensely difficult to implement frameworks for decision-makers (2).

The major topics of ED research deal with exposure, specifically BPA, and contamination of water. Accordingly, the areas of national regulatory frameworks can be distinguished in the regulation of industrial chemicals and pesticides: Environmental protection, consumer safety, and occupational health and safety. Asia now dominates global BPA capacity, with China leading the way, followed by India and South Korea (38). Research on BPA is a relatively new branch of science (39). Yet, it is the most frequently used keyword in this study. There is a lack of information on the extent of exposure to EDs, especially in developing countries, and this is also true for restrictions. However, the sparse data suggest that the exposure is similar to that in developed countries (40). Based on data from wastewater-based epidemiology, BPA exposure is extremely high in Brazil, followed at a greater distance by Japan, Germany, Canada, and China. In terms of BPA intake measured by urinary excretion, according to the study, Norway was by far the most affected country, followed by Sweden, France, Puerto Rico, Italy, and Cyprus, with less than half the amount and relatively similar exposure levels (41).

The burden of contamination with EDs is relevant for national publication efforts. The significant correlation between the proxy measure for exposure to EDs, PWG, and publication output points to this. In this context, China, Germany, and Brazil have fallen behind despite their high publication performance. That was measured using the residuals of the linear regression. As a responsibility for high emission levels, these results show that research efforts need to be intensified, even if a large publication output has already been achieved. In comparison, the ratio of items per PWG highlights those countries that perform relatively well without emitting large amounts of EDs into the environment. In this context, Denmark and Sweden rank first by a wide margin and are also far ahead in demographic indicators. These results are consistent with the efforts of individual governments to address EDs and take the threats seriously, e.g., the Danish Center on Endocrine Disruptors was established “to build and collect new knowledge on EDCs” (42). This will serve as the basis for regulations, consumer information, and criteria for ecolabels, which are urgently needed not only regionally but worldwide.

Although there have been investigations on the hazards of EDs for the last 25 years, very little awareness has reached the public (39) of what is necessary to avoid ED compounds in daily life.

In 2017, the UN reviewed existing initiatives by stakeholders from governments, civil society, and industry and identified a lack of contributions from developing countries. Industry representatives, such as the *European Crop Protection Association* (ECPA), were still calling on politicians in 2013 to consider ‘real and not potential risks’ in an attempt to weaken the demands of other interest groups (43).

Most of the political regulations and framework conditions are developed and provided by the industrialized countries, while the developing countries have few or no ED regulations (44).

US-American and European regulations have become the benchmark for other countries (33).

However, the screening and testing programs in the USA are limited only to estrogenic EDs, and EU regulations focus primarily on pesticide restriction. However, the EU also calls for minimizing human exposure to EDs in general and identifying substances of “very high concern”. This step is a start. Yet national efforts, including US action, are far too limited or nonexistent, as in many other countries. It can be argued that minimizing exposure is too slow and insufficient, especially given the many proven health effects and costs to all economies. In addition, too few substances were tested for endocrine effects (45). Especially in humans, this risk assessment is not straightforward because many confounding factors counteract or interact with the health effects of EDs. The ubiquity and unpredictability of mixtures and the likelihood of non-monotonic dose-response relationships of EDs require integrative approaches (46).

To this end, internationally networked approaches on a multidisciplinary level are required as the area ED “is – and has always been – multidisciplinary to its core” (2).

## Conclusions

The realization that endocrine disruptors are harmful to the environment and human health has led to increased research activity in most countries and thus to the identification and monitoring of more and more substances with hazardous potential. The strong North-South divide in global scientific endeavors needs to be mitigated through more networking with low- and middle-income economies. In addition, the transfer of information and knowledge to the public must be fundamentally promoted, especially in light of the immense costs that ED-related diseases impose on health systems worldwide.
